# Classification of Progression Patterns in Glioblastoma: Analysis of Predictive Factors and Clinical Implications

**DOI:** 10.3389/fonc.2020.590648

**Published:** 2020-11-03

**Authors:** Haihui Jiang, Kefu Yu, Mingxiao Li, Yong Cui, Xiaohui Ren, Chuanwei Yang, Xuzhe Zhao, Song Lin

**Affiliations:** ^1^ Department of Neurosurgery, Beijing Tiantan Hospital, Capital Medical University, Beijing, China; ^2^ National Clinical Research Center for Neurological Diseases, Center of Brain Tumor, Beijing Institute for Brain Disorders and Beijing Key Laboratory of Brain Tumor, Beijing, China; ^3^ Department of Pharmacy, Beijing Tiantan Hospital, Capital Medical University, Beijing, China

**Keywords:** glioblastoma, progression, subventricular zone, prognosis, nomogram

## Abstract

**Background:**

This study was designed to explore the progression patterns of IDH-wildtype glioblastoma (GBM) at first recurrence after chemoradiotherapy.

**Methods:**

Records from 247 patients who underwent progression after diagnosis of IDH-wildtype GBM was retrospectively reviewed. Progression patterns were classified as either local, distant, subependymal or leptomeningeal dissemination based on the preoperative and serial postoperative radiographic images. The clinical and molecular characteristics of different progression patterns were analyzed.

**Results:**

A total of 186 (75.3%) patients had local progression, 15 (6.1%) patients had distant progression, 33 (13.3%) patients had subependymal dissemination, and 13 (5.3%) patients had leptomeningeal dissemination. The most favorable survival occurred in patients with local progression, while no significant difference of survival was found among patients with distant progression, subependymal or leptomeningeal dissemination who were thereby reclassified into non-local group. Multivariable analysis showed that chemotherapy was a protective factor for non-local progression, while gender of male, subventricular zone (SVZ) involvement and O^6^-methylguanine-DNA-methyltransferase (MGMT) promoter methylation were confirmed as risk factors for non-local progression (*P* < 0.05). Based on the factors screened by multivariable analysis, a nomogram was constructed which conferred high accuracy in predicting non-local progression. Patients in non-local group could be divided into long- and short-term survivors who differed in the rates of SVZ involvement, MGMT promoter methylation and reirradiation (*P* < 0.05), and a nomogram integrating these factors showed high accuracy in predicting long-term survivors.

**Conclusion:**

Patients harboring different progression patterns conferred distinct clinical and molecular characteristics. Our nomograms could provide theoretical references for physicians to make more personalized and precise treatment decisions.

## Introduction

Glioblastoma (GBM) is the most common primary central nervous system malignancy in adults which confers a gloomy prognosis even after receiving maximal safe resection and chemoradiotherapy (1–3). More than half of patients will undergo progression at the time of six months post operation and the majority will die in 2 years (3, 4). According to the previous studies, about 80% GBM cases would suffer progression within primary treatment field (5, 6). However, patients with different progression patterns, including distant metastasis and leptomeningeal spread, have been increasingly reported in recent years (7, 8). It’s noteworthy that there is no broad consensus on the classification of patients’ progression patterns (9). Furthermore, controversy still remains regarding the clinical implication of different progression patterns (6, 7, 10).

To better address the clinical practice of patients with GBM, we should have a comprehensive understanding of predictive factors and prognostic potential of different progression patterns, especially for non-local failure. There is increasing evidence that the subventricular zone (SVZ) may be involved in the progression of GBM, because the dysregulated neural stem cells of SVZ can leave the niche and migrate over long distances, and finally contribute to the oncogenesis (11, 12). Numerous studies have shown that patients whose lesions involved the SVZ have worse clinical outcomes and more aggressive patterns of recurrence (13, 14). Moreover, those with SVZ involvement have been demonstrated to show a higher propensity to chemotherapy and radiation resistance (15). These findings have manifested the significance of the SVZ as a critical factor for GBM progression and treatment resistance.

Therefore, in this study, we retrospectively reviewed the clinical and molecular data of 247 patients who underwent the first progression after diagnosis of isocitrate dehydrogenase (IDH) wildtype GBM. To our knowledge, it presents the largest sample committed to clarifying the progression patterns of GBM after 2010 when Response Assessment in Neuro-Oncology (RANO) criteria was established (16). The primary objective of our study was to explore the role of SVZ in GBM progression and establish the prognostic significance and features of different progression patterns.

## Materials and Methods

### Patients Cohort

Records from a consecutive series of 247 patients who were diagnosed with primary GBM and experienced progression in Beijing Tiantan Hospital were retrospectively reviewed. The inclusion criteria were: pathologically diagnosed as GBM, molecular analysis showed a wildtype IDH, tumor located in supratentorial region based on the preoperative magnetic resonance (MR) images, tumor underwent progression after operation, and hospitalization from September 2011 to December 2019. The exclusion criteria were: patients received adjuvant radiotherapy or chemotherapy before resection, loss of follow-up, concurrent with other malignancies or death from other lethal diseases.

### Pathological Evaluation

For histopathological evaluation, the resected tumor tissues were fixed in 10% formalin and embedded in paraffin wax. Hematoxylin and eosin (H&E) staining and immunohistochemistry (IHC) were performed on the slices of 5 µm thick. All slices were reviewed by experienced neuropathologists according to the WHO classification system (17, 18). IHC staining was performed on a Ventana BenchMark XT autostainer (Ventana Medical System Inc, Tucson, Arizona) with antibodies against epidermal growth factor receptor (EGFR, Invitrogen), P53 (Invitrogen), and Ki-67 (Invitrogen). The specific experiment protocol and interpretation principle have been elaborated in a prior study (19). Ki-67 index was defined as either high level (≥30%) or low level (<30%) based on the percentage of IHC-positive cells (10).

In addition, telomerase reverse transcriptase (TERT) and IDH mutations were detected by Sanger sequencing using a HITACHI 3500xL Dx Genetic Analyzer (Applied Biosystems Inc, USA). The promoter region of TERT was amplified with the following primers: TERT-F, 5’-GTCCTGCCCCTTCACCTT-3’ and TERT-R, 5’-CAGCGCTGCCTGAAACTC-3’. The primers of IDH were as follows: IDH1-F, 5’-ACCAAATGGCACCATACG-3’ and IDH1-R, 5’-TTCATACCTTGCTTAATGGGG-3’; IDH2-F, 5’-GCTGCAGTGGGACCACTATT-3’ and IDH2-R, 5’-TGTGGCCTTGTACTGCAGAG-3’. Abnormality of chromosome 1p and 19q and O^6^-methylguanine-DNA methyltransferase (MGMT) promoter methylation were analyzed with fluorescence *in situ* hybridization (FISH) and pyrosequencing, respectively, according to a prior study (20) (**Figure S1**).

### Evaluation of Progression Pattern and SVZ Involvement

**Figure 1:**
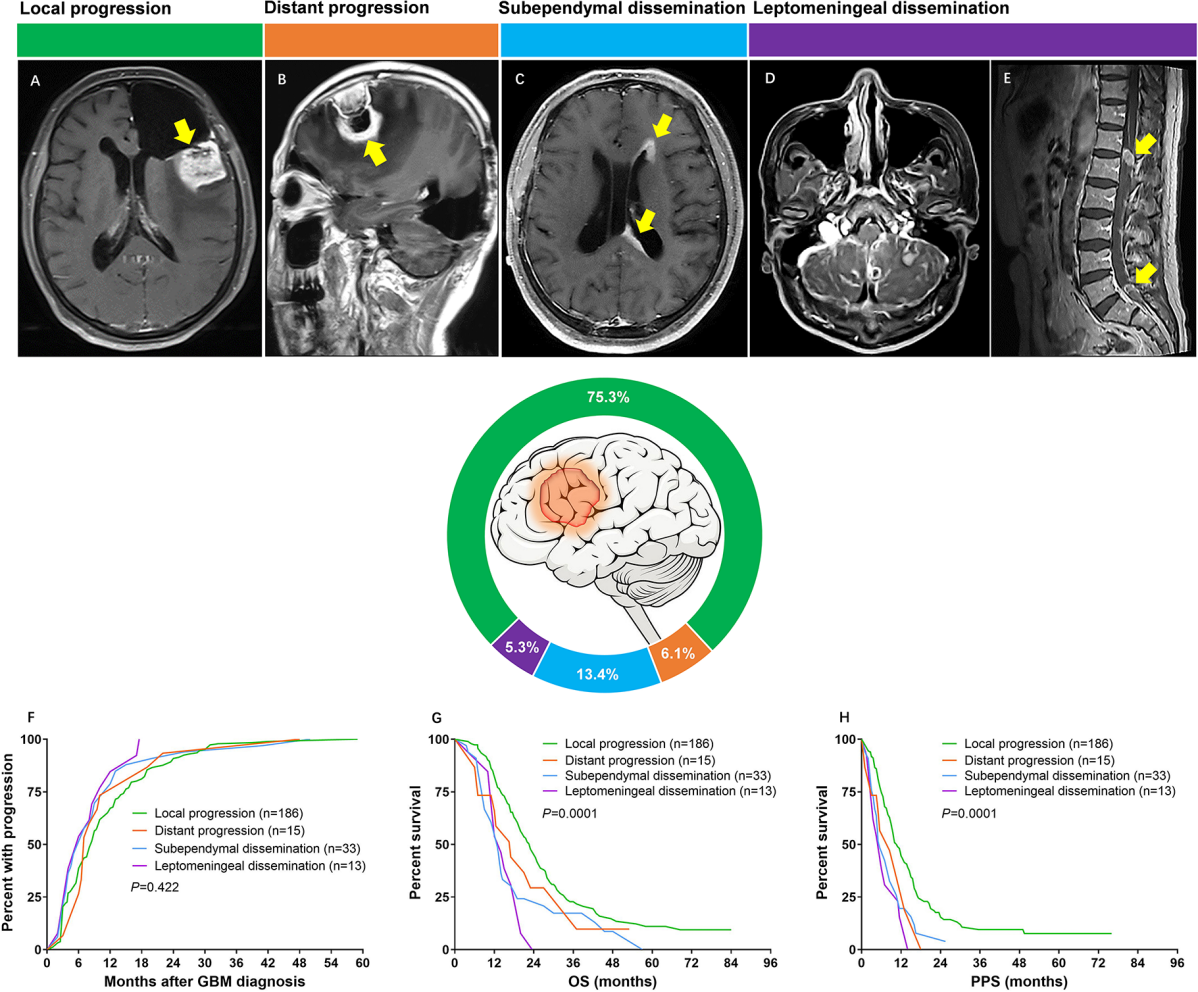
**(A)** Axial T1 image showing tumor recurred at the resection cavity (yellow arrow). **(B)** Sagittal T1 image showing a new lesion far from the original resection cavity was found (yellow arrow). **(C)** Axial T1 image showing subependymal dissemination (yellow arrows); **(D)** Axial T1 image showing superficial leptomeningeal dissemination; **(E)** Sagittal T1 image of spine demonstrating enhanced lesions (yellow arrows). **(F)** Median time from diagnosis to development of progression was 8.5 months for local progression, 7.0 months for distant progression, 6.0 months for subependymal dissemination and 6.0 months for leptomeningeal dissemination (*P* = 0.422). **(G)** Median OS was 23.0 months for patients with local progression, 17.0 months for patients with distant progression, 13.0 months for subependymal dissemination and 14.0 months for leptomeningeal dissemination (*P* = 0.0001). **(H)** Median PPS was 11.0 months for patients with local progression, 8.5 months for patients with distant progression, 5.5 months for subependymal dissemination and 6.0 months for leptomeningeal dissemination (*P* = 0.0001).

All progression patterns were analyzed by an independent review team consisted of a neuroradiologist and a neurosurgeon who were blinded to the outcome of patients. Tumor progression patterns were defined according to the following definition criteria: (1) local progression: recurrence contiguous with the resection cavity or original tumor site (**Figure 1A**). (2) distant progression: focal recurrence that was not contiguous with the resection cavity or original tumor site (**Figure 1B**). (3) subependymal dissemination: lesions disseminated along with the subependymal zone (**Figure 1C**). (4) leptomeningeal dissemination: leptomeningeal contrast enhancement around the contours of the gyri and sulci or multiple nodular deposited in the subarachnoid space. (**Figures 1D, E**). The SVZ was considered involved if tumors with enhanced lesion touching the lining of the lateral ventricle (21).

### Treatment

Once patient was radiologically diagnosed with GBM, maximal safe resection was attempted. In order to evaluate the extent of resection (EOR) of each patient, a MR was routinely performed within 48-72 hours after operation. EOR was determined according to the following equation: (preoperative tumor volume – postoperative tumor volume)/preoperative tumor volume, based on the contrast-enhanced T1 weighted imaging. Calculation of tumor volume was performed by multiplying the sum of enhanced regions outlined on each transverse slice by the corresponding slice thickness. An EOR ≥ 98% was defined as gross-total resection (GTR) (22). After operation, Stupp protocol was started in one month (3). Briefly, radiotherapy divided into 30 daily fractions of 2 Gy each was delivered to patients within 1-month post operation. Concomitant chemotherapy consisted of temozolomide (TMZ) at a dose of 75mg/(m^2^.d) was given during the whole period of radiotherapy. After a 4-week break, patients would receive 6 cycles of adjuvant TMZ at a dose of 150–200 mg/(m^2^.d), consecutive 5 days in a 28 days cycle. When tumor progressed, patients were treated at the advice of multi-disciplinary team. Reoperation was mainly considered for patients with local progression, while reirradiation was recommended to those with non-local progression. Systemic treatment could be also attempted if patients showed relatively normal laboratory tests. The most common regimen was bevacizumab (10 mg/kg in every 2 weeks) and dose-dense TMZ (100–150 mg/m^2^/d, 7 days on and 7 days off).

### Follow-Up

Patients were routinely followed up using MR scans with an interval of 3 months, or 1 month if there was any proof indicated disease progression. To improve the diagnostic accuracy of recurrence, multimodal MR including perfusion, diffusion and magnetic resonance spectroscopy was used to rule out radiation necrosis and pseudoprogression. All patients were followed until death or censored at the last follow-up. Progression represented a ≥25% increase in the maximal cross-sectional tumor area, or the appearance of any new lesion, or significant increase in T2/FLAIR nonenhanced lesions. Overall survival (OS) was defined as the time period from the date of operation to the date of death or last follow-up. Timespan between tumor progression and death/last follow-up was defined as post-progression survival (PPS). The median follow-up of this cohort was 43.0 (range: 2.0–84.0) months. All the patients experienced progression and 187 (75.7%) patients died at the time of data analysis.

### Statistical Analysis

Summary of data were presented as the mean ± SD for parametric variables and percentage for categorical variables. Comparisons of categorical variables between groups were performed using Chi-square test or Fisher’s exact test, as appropriate. Differences in age, tumor size and preoperative Karnofsky performance scale (KPS) score were evaluated by student t-test. The variables with P values less than 0.1 were entered into the multivariate logistic regression analyses to identify the predictors of non-local progression and LTS. On the basis of the predictors screened by regression analyses, nomogram models were constructed. The performance of models was evaluated by discrimination and calibration. The calibration of models was performed by a visual calibration curve comparing the predicted and actual probability. Furthermore, the nomogram models were subjected to 1000 bootstrap repetitions for internal validation to assess the predictive accuracy. The survival rate of patients was estimated with Kaplan-Meier plot, and differences between curves were compared by log-rank test. Cox proportional hazard regression model was constructed to estimate the hazard ratio (HR) for each potential prognostic factor. All data were analyzed with SPSS software package version 22.0 (IBM Corporation, Armonk, NY, USA) and R software (http://www.r-project.org). Probability values were obtained using 2-sided tests, and a *P* value of <0.05 was considered to be statistically significant.

## Results

### Demographics and Clinical Characteristics

According to the inclusion and exclusion criteria, a total of 247 patients were enrolled in the present study, including 157 (63.6%) males and 90 (36.4%) females with a mean age of 48.5 ± 11.7 years. The clinical, radiological and molecular data of our cohort were shown in **Table 1**.

**Table 1:** Comparison of baseline characteristics between different progression patterns.

Variable	Local (n = 186)	Non-local (n = 61)	*P* value
Age (years)	49.0 ± 11.2	47.2 ± 13.1	0.308
Gender (n, %)			0.056
Male	112/186 (60.2%)	45/61 (73.8%)	
Tumor size (mm)	50.1 ± 14.4	46.8 ± 16.5	0.146
Preoperative KPS	77.3 ± 15.2	75.4 ± 13.2	0.421
SVZ involvement (n, %)			**0.010**
Yes	93/186 (50.0%)	42/61 (68.9%)	
Extent of resection (n, %)			0.917
GTR	99/186 (53.2%)	32/61 (52.5%)	
Radiotherapy (n, %)			0.630
Yes	163/186 (87.6%)	52/61 (85.2%)	
Chemotherapy (n, %)			**0.028**
Yes	173/186 (93.0%)	51/61 (83.6%)	
1q polysomy (n, %)			0.152
Yes	28/158 (17.7%)	5/53 (9.4%)	
19p polysomy (n, %)			0.192
Yes	54/158 (34.2%)	13/53 (24.5%)	
MGMT promoter (n, %)			**0.016**
Methylation	59/176 (33.5%)	31/61 (50.8%)	
TERT promoter (n, %)			0.288
Mutation	42/72 (58.3%)	10/22 (45.5%)	
P53 expression (n, %)			0.533
Positive	107/157 (68.2%)	33/52 (63.5%)	
EGFR expression (n, %)			0.204
Positive	132/156 (84.6%)	40/52 (76.9%)	
Ki-67 index (n, %)			0.696
High	68/186 (36.6%)	24/61 (39.3%)	

KPS, Karnofsky performance score; SVZ, subventricular zone; GTR, gross-total resection; MGMT, O^6^-methylguanine-DNA-methyltransferase; TERT, telomerase reverse transcriptase; EGFR, epidermal growth factor receptor. In bold: p value less than 0.05.

### Patterns of Progression and Outcomes

At presentation, there were 186 (75.3%) patients with local progression, 15 (6.1%) with distant progression, 33 (13.3%) with subependymal dissemination, and 13 (5.3%) with leptomeningeal dissemination (**Figure 1**).

Time from pathological diagnosis to development of progression was similar across different patterns. Patients with GBM experienced local progression at a median period of 8.5 months, distant progression at a median period of 7.0 months, both subependymal and leptomeningeal dissemination at a median period of 6.0 months (*P* = 0.422, **Figure 1F**). In contrast, the survival of patients varied by progression patterns. The most favorable OS occurred in the group of local progression (23.0 months), followed by the group of distant progression (17.0 months), then the group of leptomeningeal dissemination (14.0 months), and tailed by the group of subependymal dissemination (13.0 months) (*P* = 0.0001) (**Figure 1G**). Similarly, the median PPS of patients with local progression, distant progression, leptomeningeal and subependymal dissemination was 11.0, 8.5, 6.0, and 5.5 months, respectively, which imparted a significant difference (*P* = 0.0001) (**Figure 1H**).

### Risk Factors for Non-Local Progression

**Figure 2:**
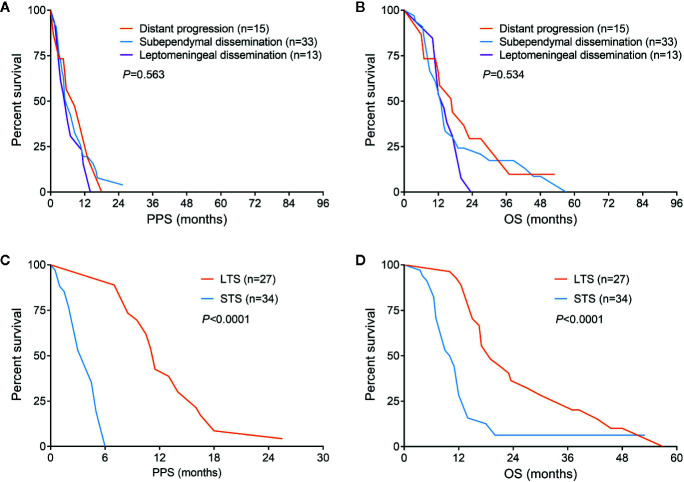
Survival comparisons among different progression patterns **(A**, **B)** and patients with non-local progression could be divided into long-term and short-term survivors **(C**, **D)**.

Considering that there was no significant difference of survival among patients with distant progression, subependymal or leptomeningeal dissemination, these patients were reclassified into non-local group for the convenience of comparative analysis (**Figures 2A, B**). According to the results of intergroup comparisons, we found male was slightly more common in non-local group comparing with those in local group (*P* = 0.056). The frequency of SVZ involvement in local group was significantly lower than that in non-local group (50.0 vs. 68.9%, *P* = 0.01). Most of the treatments were equivalent between the two groups, except patients with non-local progression had a lower rate of chemotherapy (83.6 vs. 93.0%, *P* = 0.028). With respect to the molecular data, MGMT promoter methylation could be found in 31 of 61 patients with non-local progression, which was higher than that in patients with local progression (50.8 vs. 33.5%, *P* = 0.016) (**Table 1**).

**Figure 3:**
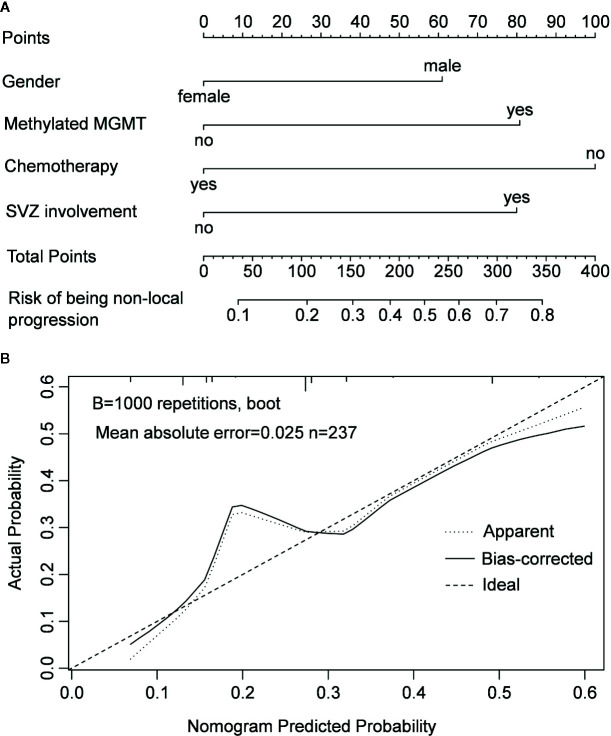
Nomogram model for predicting the risk of being non-local progression in patients with IDH-wildtype GBM **(A)** and calibration curve of the nomogram model **(B)**.

In the multivariable logistic regression analysis, chemotherapy was the only protective factor of non-local progression (odds ratio [OR] = 0.316, 95% CI: 0.122–0.814, *P* = 0.017). Meanwhile, gender of male, SVZ involvement, and MGMT promoter methylation were confirmed as risk factors for non-local progression (OR = 2.020, 95% CI: 1.018–4.007, *P* = 0.044; OR = 2.516, 95% CI: 1.317–4.805, *P* = 0.005 and OR = 2.539, 95% CI: 1.352–4.768, *P* = 0.004, respectively) (**Table 2**). A nomogram model that integrated these independent factors was constructed. We could estimate the risk of patient developed to non-local progression after operation by adding the score of each factor which was shown in **Figure 3A**. The concordance index (C-index) for the prediction nomogram was 0.88. Calibration curve demonstrated excellent agreement between predicted and observed probability of non-local progression (**Figure 3B**).

**Table 2:** Results of multivariate logistic regression analysis.

Variables	Odds ratio	95% Confidence interval	*P* value
Predictors for non-local progression			
Gender (male)	2.020	1.018-4.007	**0.044**
SVZ involvement (yes)	2.516	1.317-4.805	**0.005**
Chemotherapy (yes)	0.316	0.122-0.814	**0.017**
MGMT promoter (methylation)	2.539	1.352-4.768	**0.004**
Predictors for LTS in non-local group			
SVZ involvement (yes)	0.124	0.022-0.690	**0.017**
MGMT promoter (methylation)	5.506	1.271-23.851	**0.023**
Reirradiation (yes)	5.238	1.106-24.807	**0.037**

SVZ, subventricular zone; MGMT, O^6^-methylguanine-DNA-methyltransferase; LTS, long-term survivors. In bold: p value less than 0.05.

### Long- and Short-Term Survivors in Patients With Non-Local Progression

**Figure 4:**
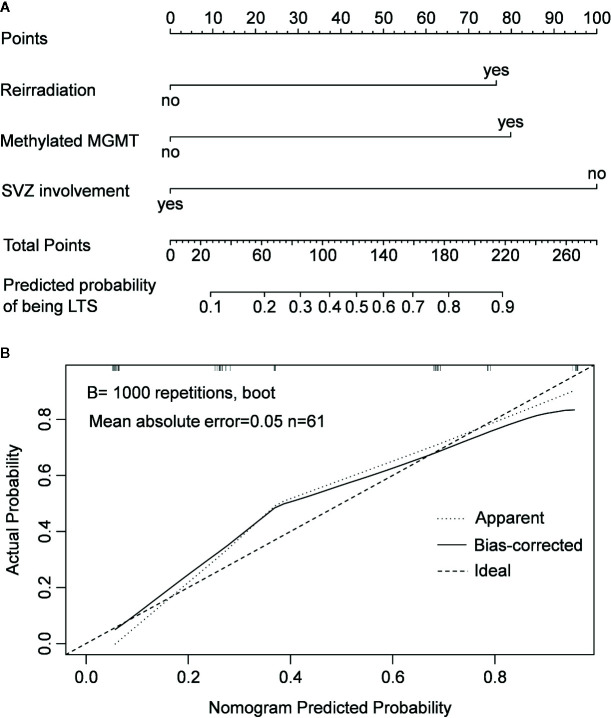
Nomogram model for predicting the probability of being long-term survivors in patients with non-local progression **(A)** and calibration curve of the nomogram model **(B)**.

Since patients showed a median survival of 6.0 months after diagnosis of non-local progression, they were divided into long-term survivors (LTS) and short-term survivors (STS) at a cutoff of 6.0 months (**Figures 2C, D**). Compared with STS, LTS had a lower rate of SVZ involvement (44.4 vs. 88.2%, *P* < 0.001) but higher rates of reirradiation (70.4 vs. 32.4%, *P* = 0.003) and MGMT promoter methylation (81.5 vs. 26.5%, *P* < 0.001) (**Table 3**). In the multivariable analysis, all these three parameters were further confirmed as independent predictors of LTS (*P* < 0.05) (**Table 2**). According to these predictors, we built a nomogram model to predict the probability of being LTS. This model conferred a C-index of 0.70 (**Figure 4A**). The calibration curve presented a good agreement between the prediction based on our nomogram and actual observation (**Figure 4B**).

**Table 3:** Comparison of baseline characteristics between long- and short-term survivors in non-local group.

Variable	Long-term survivors (n = 27)	Short-term survivors (n = 34)	*P* value
Age (years)	49.3 ± 14.1	45.5 ± 12.2	0.256
Gender (n, %)			0.071
Male	23/27 (85.2%)	22/34 (64.7%)	
Tumor size (mm)	43.0 ± 16.0	50.0 ± 16.5	0.101
KPS score	72.9 ± 14.6	77.3 ± 12.1	0.223
SVZ involvement (n, %)			**<0.001**
Yes	12/27 (44.4%)	30/34 (88.2%)	
Extent of resection (n, %)			0.933
GTR	14/27 (51.9%)	18/34 (52.9%)	
Reirradiation (n, %)			**0.003**
Yes	19/27 (70.4%)	11/34 (32.4%)	
Re-chemotherapy (n, %)			0.482
Yes	19/27 (70.4%)	21/34 (61.8%)	
Yes	2/22 (9.1%)	5/31 (16.1%)	
1q polysomy (n, %)			1.0*
Yes	2/22 (9.1%)	3/31 (9.7%)	
19p polysomy (n, %)			0.092
Yes	8/22 (36.4%)	5/31 (16.1%)	
MGMT promoter (n, %)			**<0.001**
Methylation	22/27 (81.5%)	9/34 (26.5%)	
TERT promoter (n, %)			0.383*
Mutation	2/7 (28.6%)	8/15 (53.5%)	
P53 expression (n, %)			0.117
Positive	16/21 (76.2%)	17/31 (54.8%)	
EGFR expression (n, %)			1.0*
Positive	16/21 (76.2%)	24/31 (77.4%)	
Ki-67 index (n, %)			0.210
High	13/27 (48.1%)	11/34 (32.4%)	

SVZ, subventricular zone; KPS, Karnofsky performance scale; GTR, gross-total resection; MGMT, O^6^-methylguanine-DNA-methyltransferase; TERT, telomerase reverse transcriptase; EGFR, epidermal growth factor receptor.

*Fisher’s-Exact Test. In bold: p value less than 0.05.

### Multivariate Survival Analysis

**Figure 5:**
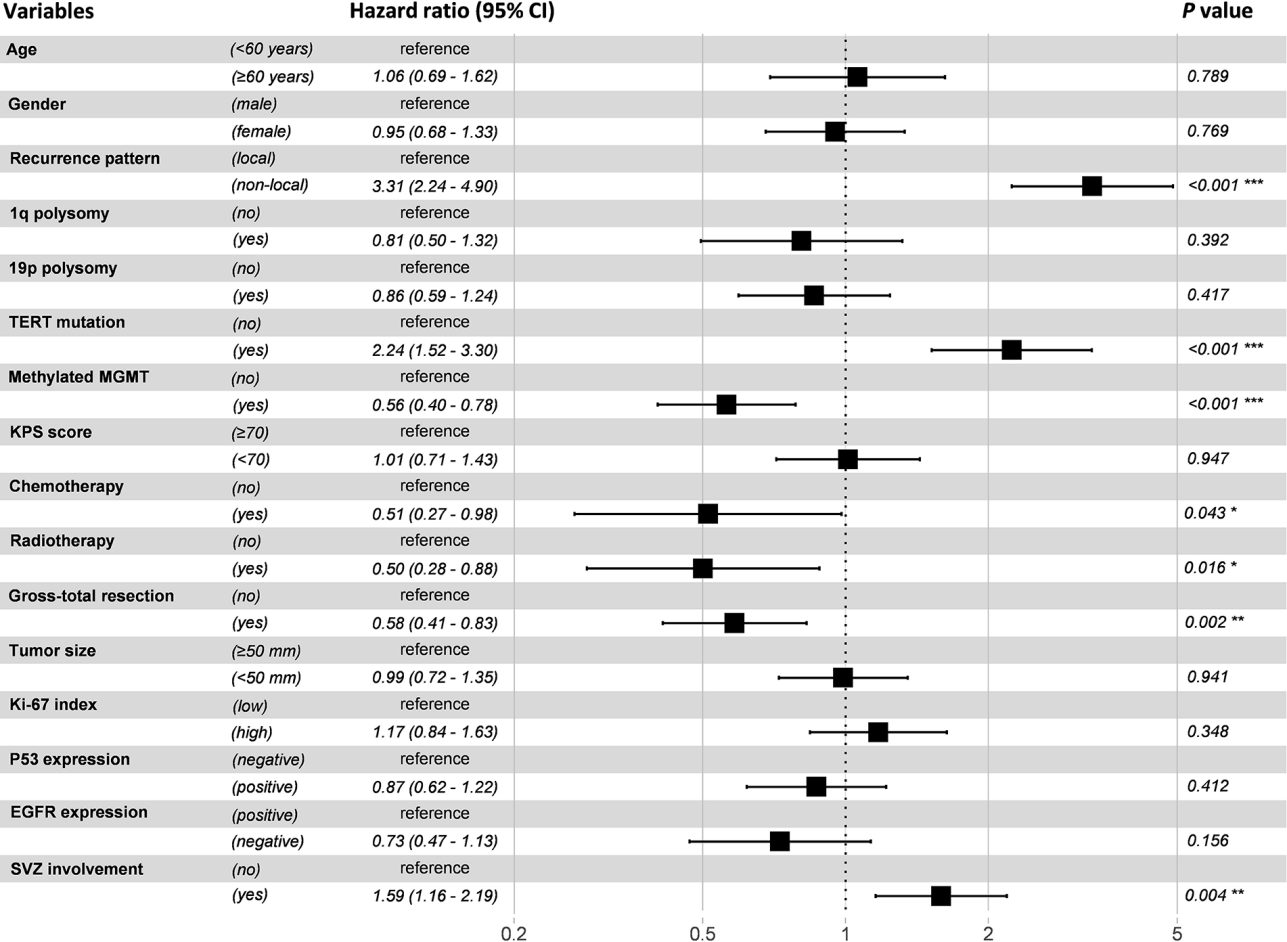
Forest plot of multivariable Cox proportional hazard regression analysis. Variables with a hazard ratio larger than 1 were considered as risk factors, while those with a hazard ratio less than 1 were considered as protective factors.

A Cox proportional hazard regression model including all recorded potential prognostic factors was established. The results showed that progression pattern, TERT promoter mutation, MGMT promoter methylation, chemotherapy, radiotherapy, GTR, and SVZ involvement were identified as independent prognostic factors (*P* < 0.05) (**Figure 5**).

## Discussion

This study systematically elucidated the incidence and implication of different progression patterns in GBM population. We reclassified the progression pattern into two subtypes: local and non-local, which could be easily applied in routine clinical practice. In addition, the predictive factors of non-local progression and LTS have also been identified. Furthermore, nomogram models were constructed which could predict the risk of being non-local progression in patients with IDH-wildtype GBM and estimate the probability of being LTS in non-local group.

Tumor progression seems to be an inevitable outcome in terms of the current treatment status of GBM. Although the majority of GBM cases suffer local failure, non-local progression can be also encountered in clinical practice and still shows a clear upward trend in recent years (5–8, 23). The reported rate of non-local progression of GBM at first recurrence ranges from 2% to 34.5% (5–8, 24–31). In the present study, 61 (24.7%) patients developed non-local progression which was in accordance with prior studies (28, 30, 31). The variable incidence of different progression patterns is significantly correlated with classification criteria. However, no definite consensus on the definition of progression patterns so far has been reached (9). Several studies divided the progression patterns of GBM into four types: local, diffuse, distant and multifocal (5, 27). The most common definition for local progression is recurrence contiguous with the resection cavity or original tumor site (9). There are also reports delineated local failure according to the distance between recurred lesion and original resection cavity (9, 27, 32–34). But the distance is not uniformly deﬁned in different studies (27, 32–34). Moreover, the definition of diffuse progression varies greatly. Piper et al. even state that the diffuse progression is a kind of pattern being poorly understood (9), while we think both local and diffuse progression is the same pattern which has been detected in different time period.

The impact of progression patterns on survival is still controversial (6, 10, 29, 35–37). No significant difference has been observed in terms of the time to development of different progression patterns in this study, which was consistent with prior reports (10, 36). But Tejada et al. held that the median progression-free survival of patients underwent non-local failure was signiﬁcantly longer than those with local failure (29). However, we found that the OS and PPS of patients were highly dependent on progression patterns. The most favorable prognosis occurred in local group, while no significant difference of survival was observed among patients with distant progression, subependymal or leptomeningeal dissemination. This survival advantage of local progression over non-local progression may partly result from the higher rate of reoperation (29.6 vs. 16.4%, *P* = 0.043). Brandes et al. reported that patients with recurrence out of radiation ﬁeld conferred a longer survival than those showing recurrence within radiation ﬁeld, which was attributed to the improved local control (6). However, non-local failure is commonly considered as a sign of advanced stage of GBM and indicates a worse prognosis (7, 10, 35). Therefore, considering the contradictory results on the definition and implication of progression patterns, we divided the progression pattern of GBM into local and non-local subtypes based on clear and easily replicable criteria, which could contribute to addressing these controversies.

We also explored factors that predisposed to the development of non-local progression in this study. In agreement with previous studies, MGMT promoter methylation and gender of male were found to be predictors of non-local progression (6, 38, 39). Methylated MGMT promoter was associated with higher chemosensitivity, which would lead to an improved local control for patients with GBM. It has also been confirmed by our Cox regression model (**Figure 5**). While male patients presented a higher risk for non-local progression might be ascribed to the unfavorable response to treatment (40). Additionally, SVZ involvement was identified as an independent risk factor of non-local progression in our study. This finding is in congruence with Lim et al. and Adeberg et al., who found an association between neurogenic niche contact, multifocal distant progression, and poor outcome (13, 21). SVZ is regarded as a neurogenic region where has been resided by neural stem cells. It is hypothesized that GBM cancer stem cells may stem from dysregulated neural stem cells (41). Given that tumors involved SVZ are in close proximity with cerebrospinal fluid (CSF), our finding can be explained by the hypothesis that CSF circulation may seed tumor cells to distant sites. This hypothesis has been proved by Shibahara et al. who found a high CD133 expression in patients with distant failure (42). Fortunately, chemotherapy can eliminate the tumor cells in the CSF and decrease the incidence of non-local failure, which has been confirmed by our results and previous studies (10, 43).

Other factors that associated with the incidence of non-local progression has also been reported, such as radical resection (44), gains of 1p36 (45), and high epidermal growth factor receptor (EGFR) expression (46). Despite prior report suggested radical resection could influence the progression pattern of GBM by improving local control (44), we found no difference in the rate of GTR between local and non-local groups. Korshunov et al. concluded that gains of 1p36 were correlated with leptomeningeal dissemination of supratentorial GBM, which might be resulted from the activation of potent oncogenes or growth-regulating genes located in this chromosome region (45). Tini et al. illustrated that patients with high EGFR expression showed a higher rate of distant recurrence (46). But we did not find this trend in our cohort.

Considering non-local progression is an increasingly prominent clinical problem, we explored the features of LTS in non-local group. Final results demonstrated that tumor bearing SVZ involvement was associated with poor prognosis, while reirradiation played a vital role in prolonging the survival of patients with non-local progression. It supported the finding by Dardis et al. who concluded that radiotherapy could improve the OS of patients with leptomeningeal metastases (47). Interestingly, the rate of MGMT promoter methylation which has been identified as a predictor of non-local progression was remarkably increased in the subgroup of LTS. In order to disclose the potential reason for this phenomenon, we compared the survival of patients receiving re-chemotherapy or not based on the status of MGMT. Results showed that patients with methylated MGMT might benefit from re-chemotherapy, while this survival advantage disappeared when analysis focused on those with unmethylated MGMT (**Figure S2**). Therefore, re-chemotherapy seems to be a treatment option for patients with methylated MGMT when tumor underwent non-local progression.

Limitations do exist in this study. Firstly, it’s a single-center, retrospective study which inevitably includes bias in patient selection. Secondly, the MR based definition of tumor progression may imply a misinterpretation of pseudoprogression, which can, to some extent, impact the result of this study. Additionally, although internal validation of our models confers optimal discrimination and excellent calibration, the generalizability of these nomograms still requires external validation based on additional database. Finally, as the data is collected from adult neuro-oncology department, the mean age at diagnosis of GBM in this study seems to be younger than the reported data (18). Thus, our results may not be applicable for the older patients.

## Conclusions

To summarize, this study systematically analyzed the incidences, characteristics, and prognoses of different progression patterns based on a relatively larger cohort of GBM. Despite some inevitable limitations, our results suggest that gender of male, SVZ involvement and MGMT promoter methylation are correlated with higher risk for non-local progression. Our nomogram models could be used to predict the risk of being non-local progression in patients with IDH-wildtype GBM and estimate the probability of being LTS in non-local group.

## Data Availability Statement

All data supporting the conclusions of this article are available on request from any qualified investigator.

## Ethics Statement

The studies involving human participants were reviewed and approved by Institutional Review Board of Capital Medical University. The patients/participants provided their written informed consent to participate in this study.

## Author Contributions

Acquisition of data: HJ, ML, CY, and XZ. Analysis and interpretation of data: HJ, KY, YC, and XR. Statistical analysis: HJ and KY. Drafting the article: HJ and SL. Funding acquisition: SL and YC. Conception and design: HJ and SL. Study supervision: SL. All authors contributed to the article and approved the submitted version.

## Funding

This work was supported by the National Natural Science Foundation of China (81771309) and the Capital’s Funds for Health Improvement and Research (2020-2-1075).

## Conflict of Interest

The authors declare that the research was conducted in the absence of any commercial or financial relationships that could be construed as a potential conflict of interest.

## Supplementary Material

Supplementary Figure 1 Panel I, detection result of chromosome 1p and 19q: 1p intact **(A)**, 1p deletion **(B)**, 1q polysomy **(C)**; 19q intact **(D)**, 19q deletion **(E)**, and 19p polysomy **(F)**. Panel II: Wildtype IDH1 and IDH2. Panel III: MGMT promoter unmethylation **(A)** and MGMT promoter methylation **(B)**.

Supplementary Figure 2 In the subgroup of MGMT promoter methylation, patients with re-chemotherapy showed a trend toward better PPS comparing with those without re-chemotherapy (11.5 vs. 8.5 months, *P* = 0.079) **(A)**. While in the subgroup of MGMT promoter unmethylation, this trend disappeared (4.8 vs. 3.0 months, *P* = 0.232) **(B)**.
