# Supplementary figures and images for: Classification of Progression Patterns in Glioblastoma: Analysis of Predictive Factors and Clinical Implications
Source: Front Oncol. 2020 Nov 3;10:590648. doi: 10.3389/fonc.2020.590648 (PMC7673412; doi:10.3389/fonc.2020.590648)

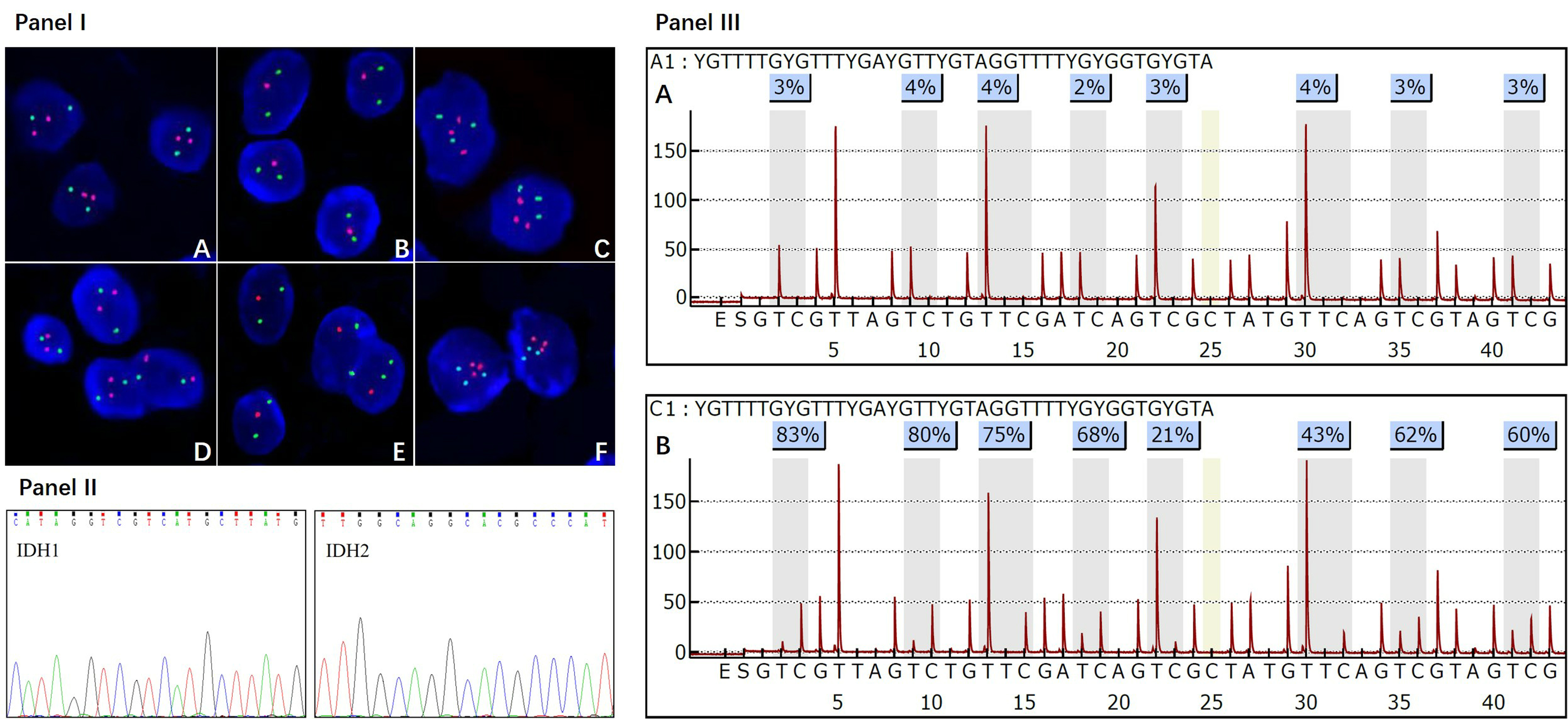

Supplement: Supplementary Figure 1 — Panel I, detection result of chromosome 1p and 19q: 1p intact (A), 1p deletion (B), 1q polysomy (C); 19q intact (D), 19q deletion (E), and 19p polysomy (F). Panel II: Wildtype IDH1 and IDH2. Panel III: MGMT promoter unmethylation (A) and MGMT promoter methylation (B). [file Image_1.jpeg]

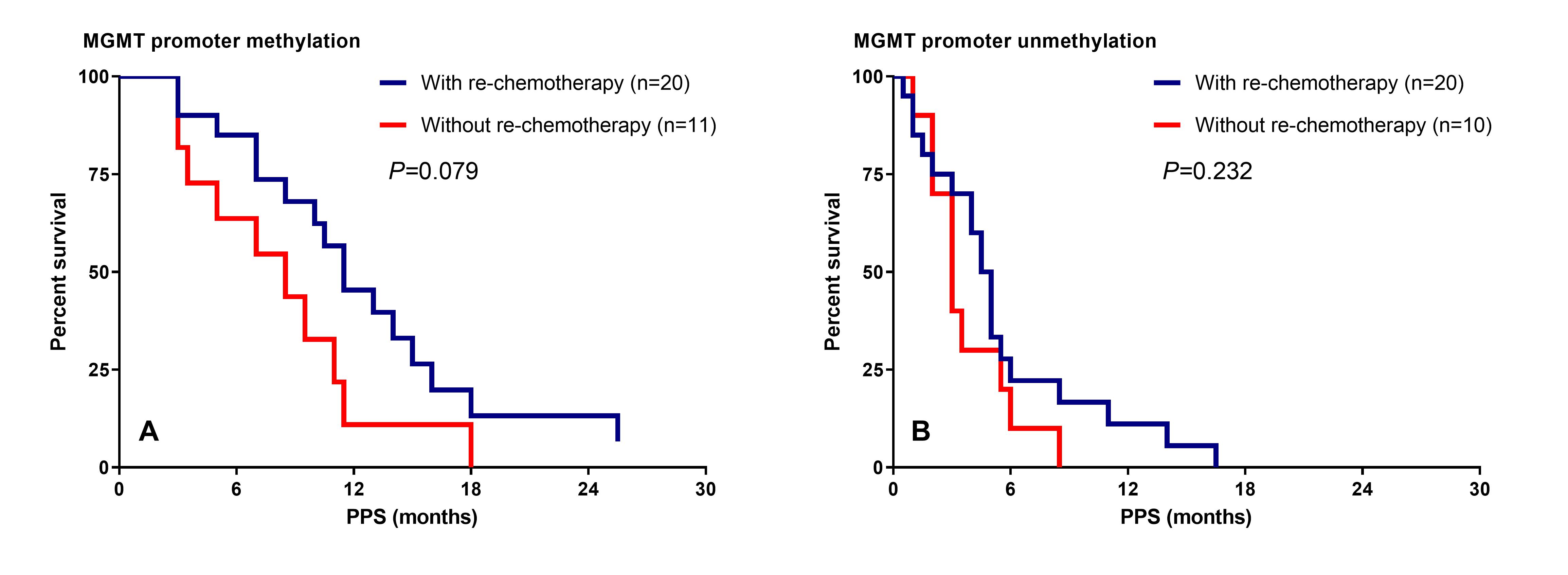

Supplement: Supplementary Figure 2 — In the subgroup of MGMT promoter methylation, patients with re-chemotherapy showed a trend toward better PPS comparing with those without re-chemotherapy (11.5 vs. 8.5 months, P = 0.079) (A). While in the subgroup of MGMT promoter unmethylation, this trend disappeared (4.8 vs. 3.0 months, P = 0.232) (B). [file Image_2.jpeg]
